# Transcutaneous ultrasonography for visualization of the kidneys in captive Asian elephants (*Elephas maximus*): a quantitative assessment of echogenicity and echotexture in comparison with the liver and spleen

**DOI:** 10.1186/s12917-025-04835-4

**Published:** 2025-05-26

**Authors:** Pratthana Inthawong, Somkiat Huaijantug, Tithipong Plangsangmas, Kakanang Piyarungsri, Taweepoke Angkawanish, Warangkhana Langkaphin, Worapong Kosaruk, Choenkwan Pabutta, Supatta Kijpraiboon, Mark A. Mitchell, Podjana Wattananit, Chatchote Thitaram

**Affiliations:** 1 Center of Elephant and Wildlife Health, Faculty of Veterinary Medicine, Chiang Mai University, Canal Road, Chiang Mai, 50100 Thailand; 2 Department of Clinical Science and Public Health, Faculty of Veterinary Science, Mahidol University, Salaya, Nakhon Pathom, 73170 Thailand; 3 Department of Veterinary Clinical Sciences, School of Veterinary Medicine, Louisiana State University, Baton Rouge, LA 70803 USA; 4 Chulabhorn Royal Academy, Lak Si, Bangkok, 90210 Thailand; 5 Faculty of Veterinary Medicine, Chiang Mai University, Canal Road, Chiang Mai, 50100 Thailand; 6 The Thai Elephant Conservation Center, National Elephant Institute of Thailand, The Forest Industry Organization, Lampang, 52190 Thailand; 7 The Monitoring and Surveillance Center for Zoonotic Diseases in Wildlife and Exotic Animals, Faculty of Veterinary Science, Mahidol University, Salaya, Nakhon Pathom, 73170 Thailand; 8 Elephant, Wildlife and Companion Animals Research Group, Chiang Mai University, Muang, Chiang Mai, 50200 Thailand

**Keywords:** Asian elephant, Gray-level histogram, Kidney, Transcutaneous ultrasound

## Abstract

**Background:**

Kidney transcutaneous ultrasonography can be used to assess renal condition and is less invasive than transrectal ultrasonography, which typically requires intensive restraint, sedation, or general anesthesia. To date, this less invasive technique has not been evaluated in Asian elephants (*Elephas maximus*). The gray level histogram technique associated with transcutaneous ultrasonography is a quantitative approach to objectively measure echogenicity and echotexture. This study utilized gray-level histograms (GLH) to assess echogenicity and echotexture of the kidneys, spleen, and liver of 49 captive Asian elephants via transcutaneous ultrasonography, to obtain a baseline for healthy animals and to compare various internal organs as a reference for quantitative analyses.

**Results:**

Retroperitoneal fat was the most hyperechoic region identified, followed by the spleen. The renal medullas and the left cortex were the three most homogenous tissues. No significant differences were found between the sexes or age groups.

**Conclusions:**

This study found that transcutaneous ultrasonography could be used to quantitatively measure echogenicity and echotexture in captive Asian elephants using the GLH technique. Baseline GLH references were developed for healthy captive Asian elephants for renal, hepatic and splenic transcutaneous ultrasonography.

## Background

Asian elephants (*Elephas maximus*) are classified as endangered species (endangered; EN) by the International Union for Conservation of Nature Red List [1] and are listed under Appendix 1 of the Convention on International Trade in Endangered Species of Wild Fauna and Flora [2]. In Thailand, there are approximately 3,100–3,600 free-living elephants in 69 protected areas and 3,783 captive elephants [3]. Data from the National Elephant Institute, Forest Industry Organization, Lampang, Thailand (T. Angkawanish, pers. comm.) revealed a high incidence of acute and chronic renal failure diagnosed from blood chemistry testing and necropsies. Renal failure, pyelonephritis and ureteral dilation were identified in affected elephants [4, 5].

Elephant kidneys are multilobulated oval-shaped organs. One kidney has an average of eight (8 ± 2) dorsoventrally symmetrical lobes. The caudal region is wider than the cranial region. The pelvis divides at the sinus into primary branches, or infundibula, which send a secondary branch, or infundibulum, into each lobe [5]. The renal cortex accounts for 73% of the kidney, while the renal medulla accounts for the remaining 27% [6]. In a study of four Asian elephant kidneys obtained from necropsy, the size of the kidneys increased with age and had lengths of 135 mm in an aborted fetus, and 170, 320, and 360 mm elephants that were 9.2 months, 19.75 and 51 years-old, respectively. Similar to ruminants, the left kidney is located more caudal than the right kidney [5].

Ultrasonography has become an essential imaging tool for identifying renal diseases in human and veterinary medicine. Ultrasonography, in addition to renal biomarkers, is commonly used to diagnose renal disease in dogs and cats by analyzing the shape, size and echogenicity of the renal pelvis, medulla and cortex [7]. Moreover, research supports the idea that renal echogenicity is a useful parameter for assessing renal function in chronic kidney disease (CKD) in human patients [8]. In domestic animals, comparison of echogenicity between the renal cortex, renal medulla, spleen, and liver was reported in clinically healthy animals [9–11].

In elephants, ultrasound images are commonly used to diagnose diseases of different systems [5]. Generally, two types of ultrasound methods are used in elephants: transcutaneous and transrectal. Transcutaneous ultrasound is commonly used for diagnosing late pregnancy, bone and joint diseases, and assessing the heart, teeth, eyes, cranial abdomen and mammary glands. Pachyderms have a thick skin, with an average depth of 22 millimeters (range 18–32 millimeters) [12]; therefore, the frequency of the ultrasound transducer has to be limited to 2–4 MHz. Transrectal ultrasound is used to evaluate the reproductive, urinary and intestinal organs using a frequency range of 2–10 MHz. However, this method is limited due to requiring sedation [13]. Several reports have documented the use of transrectal ultrasonography to assess the reproductive tract and support diagnosis and monitoring of pyelonephritis and CKD in elephants [4, 14, 15]. Hildebrandt et al. [15] performed standing sedation for transrectal ultrasonography and found that the distance from the rectum to the kidneys was 1.5–2 m, which required an extension probe for the ultrasound transducer. In these cases, only the left kidney was fully visible. Elephant kidneys from ultrasound images revealed the renal capsule, interlobar septum, caly, and secondary infundibulum as hyperechoic lines. The renal cortex appeared more echogenic than the medulla, which appeared as a hypoechoic triangular area [16]. While transrectal ultrasonography appears effective in obtaining information on mainly the left renal parenchyma imaging in Asian elephants, the necessity for additional tools and physical or chemical restraint renders this technique impractical for routine clinical use.

Measuring echogenicity by plain sight may cause misinterpretation of the renal structures as a result of different factors, including when multiple lesions are present in the tissue, the amount of fat in a tissue, and the capacity of the human eye to distinguish grayscale. To reduce the bias caused by visual assessment, an image analysis program can be used to assess the echogenicity of tissues. The software is capable of analyzing more than 255 different color shades that aim for mean gray value or mean gray level or analyze gray-level histograms (GLH) on the area of focus [11, 17–19]. The GLH technique can therefore be used to analyze the echogenicity and echotexture of tissues. The technique analyzes the quantity and level of gray color in the area of interest and uses descriptive statistics to create a histogram to view the distribution [10, 19]. Previous studies using this technology in animals include the assessment of the renal cortex in rabbits [20], evaluation and assessment of the nuchal ligament [21] and common carotid arteries [22] of horses, comparison of the kidneys, liver and spleen of horses and mules [11], staging hepatic lipidosis in cattle [23], comparing renal vs. hepatic ultrasonography in dogs [10], and correlating findings for renal histopathology and echogenicity in dogs and cats [17].

Based on the authors’ knowledge, transcutaneous ultrasound to assess renal health in Asian elephants has not been previously described. Therefore, we aimed to utilize transcutaneous ultrasound as a novel tool to assess the kidneys of Asian elephants. The objectives of this study were (1) to quantify normal echogenicity and echotexture of the kidneys, liver and spleen in Asian elephants by using the GLH technique, and (2) to compare differences of the obtained values from each region between age classes and sex. We hypothesized that (1) the echogenicity of the renal cortex would be more echogenic than the medulla, (2) the kidneys, liver and spleen would differ in echogenicity from being more hypoechoic to hyperechoic, respectively, and (3) the echogenicity and echotexture of the kidneys, liver, and spleen of elephants without evidence of renal disease would not be different between individuals, age classes and sex.

## Materials and methods

### Ethical approval

This study was approved by the Faculty of Veterinary Science, Mahidol University-Institutional Animal Care and Use Committee (MUVS-2021-08-30).

### Study period and location

The study was conducted from February 2022 to October 2022 at the Thai Elephant Conservation Center, National Elephant Institute of Thailand, Forest Industry Organization, Lampang, Thailand.

### Sample population

Forty-nine Asian elephants without evidence of renal disease were included in this study, based on a clinical examination, complete blood count and serum biochemistry. Using published reference ranges, elephants with both CBC and biochemistry values within normal range were included in this study [24]. Out of 49 elephants, 18 were females and 31 were males, with an average age of 36.23 ± 19.98 years old (range 9 to 68 years old) (juvenile and sub-adults [< 15 years] *n* = 9, adults [15–40 years] *n* = 18, and geriatric elephants [> 40 years] *n* = 22). The average body weight was 3,037.35 ± 593.81 kg (range 1,665 to 4,460 kg). The median (IQR) (range) of BCS were 3.5 (3.5-4) (2.5-4) [25]. All elephants were housed and fed under the same conditions at the Thai Elephant Conservation Center, National Elephant Institute of Thailand, Forest Industry Organization, Lampang, Thailand (latitude and longitude: 18° 17’ 53” N / 99° 30’ 25” E). Transcutaneous ultrasounds of the liver, spleen, retroperitoneal fat and kidneys were performed on all elephants.

### Ultrasonography

**Fig. 1:**
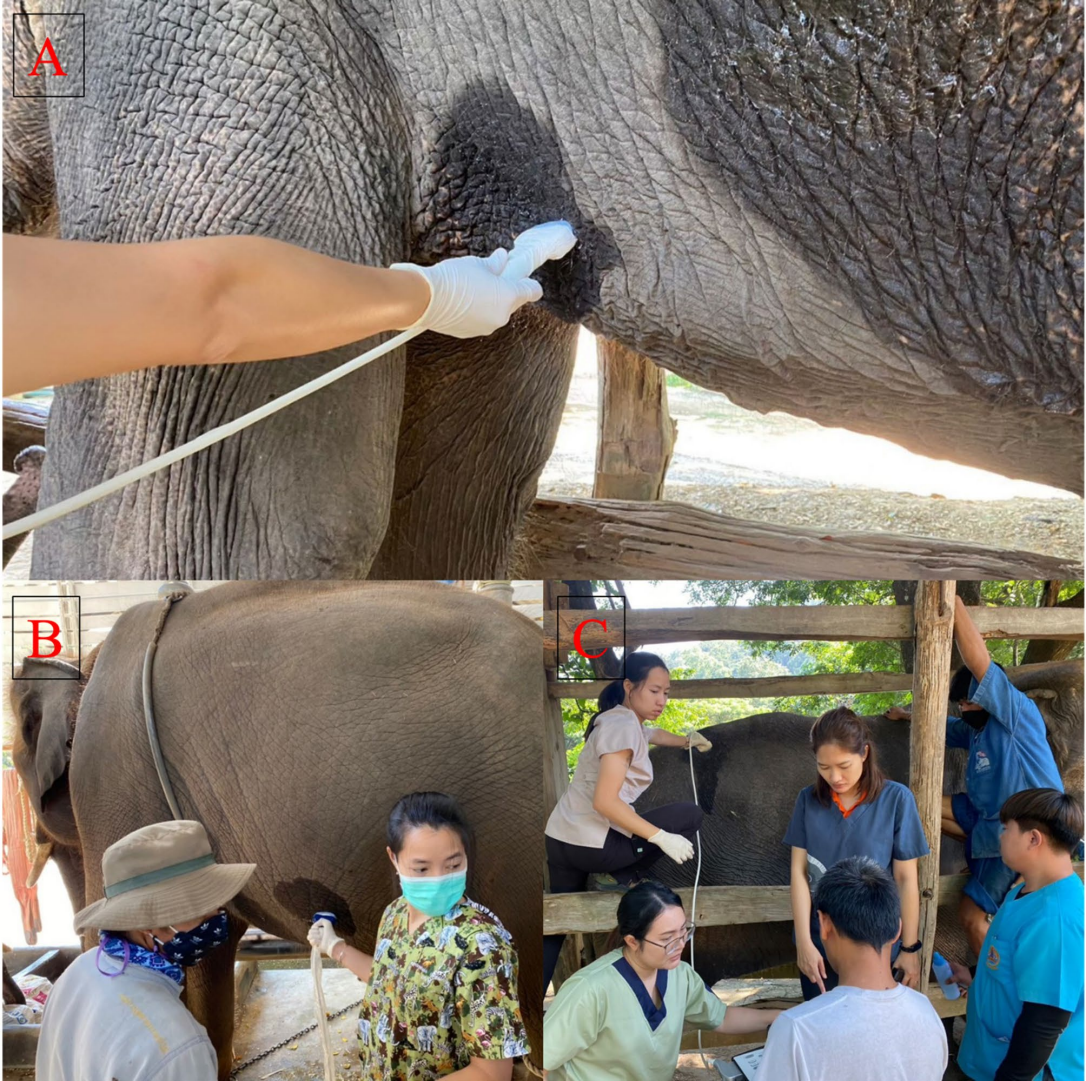
Ultrasonographic examination of the liver (**A**), spleen (**B**) and right kidney (**C**) in captive Asian elephants

Ultrasonic examinations were performed on non-sedated animals in a free contact setting without using topical or local anesthesia, and fasting was not performed. All elephants were behaviorally trained with a handler (mahout) assisting throughout the whole procedure. A mobile ultrasound (GE Logiq-e^®^ Portable; Ultrasound Machine, GE Healthcare, Chicago, IL, USA) equipped with a multifrequency convex transducer (2–5 MHz) was used to capture the images. All regions were examined at equal depth ranges (15–30 cm) centered at the focal zone on each image. Due to time constraints, ultrasonographic imaging of the liver and spleen was only conducted from the left side of elephants; the kidneys were investigated from both sides. The ultrasonographic examination of the liver was conducted in the area caudal to the left forelimb (Fig. 1A), the spleen was imaged at the level of the left central abdomen (Fig. 1B), and the kidneys were imaged at the level of the paralumbar region (Fig. 1C). The left kidney is caudal to the right kidney, and this was accounted for during image capture [6]. The location for probe placement was measured for the kidneys. The hair in the examination area was clipped, and debris was washed with clean water. A 70% isopropyl alcohol solution and ultrasound transmission gel (BM2, Anji Sunlight Medical Products Co., Ltd., Huzhou, Zhejiang, China) were applied to improve conductivity. Longitudinal and transverse images were captured using constant frequency, depth and gain (2–5 MHz, 15–30 cm, and 50–70%). The images were saved in JPEG format, and each examination lasted no longer than 30 min.

### Gray-level histogram (GLH)

**Fig. 2:**
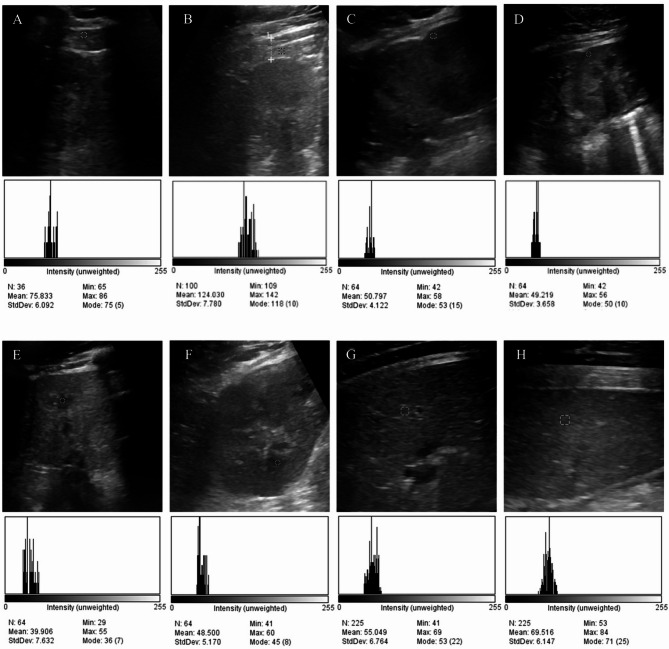
Ultrasound images and the respective gray-level histogram of the retroperitoneal fat of the right kidney (**A**) retroperitoneal fat of the left kidney (**B**), right renal cortex (**C**), left renal cortex (**D**), right renal medulla (**E**), left renal medulla (**F**), liver (**G**), and spleen (**H**). Yellow dotted lines delineate the region of interest

All sonographic images were independently evaluated by two investigators. Only images with adequate definition of the parenchyma and cortical-medullar junction were used for the GLH. For the liver, spleen, retroperitoneal fat, renal cortex and medulla evaluation, three square regions of interest (ROI) of 0.3–0.5 cm^2^ (500–1000 pixels) were selected in each region, avoiding extremities or other renal structures, such as the renal capsule, the cortical-medullar junction, diverticula or vessels (Fig. 2). A GLH analysis was performed using ImageJ software version 1.51k (National Institutes of Health, Bethesda, MD, USA), with a gray scale of 255 shades, with 0 as black pixels and 255 as white pixels. The average of the following quantitative parameters was obtained from all three ROIs: the number of pixels (COUNT); the most frequent gray tonality (MODE); the number of pixels with the MODE value (MODE COUNT); and the pixels’ mean (MEAN). The MEAN and MODE variables were used to analyze the ROI’s echogenicity as in “hypo”, “hyper”, or “iso” echoic. The ratio between MODE COUNT and COUNT (rMC: C) of each ROI was calculated to evaluate the echotexture or homogeneity as in homogenous or heterogenous for comparison between regions.

### Statistical analysis

Statistical analysis was performed using SPSS version 29.0 (IBM Statistics, Armonk, NY, USA) with statistical significance set at *P* < 0.05. MEAN, MODE, rMC: C, and their residuals were tested for normality with the Shapiro-Wilk normality test, skewness, kurtosis, Q-Q plot and histograms. Non-parametric data were log-transformed and re-tested for normality. The descriptive statistics were used to define the mean, SD, median, interquartile range (25–75%) and min-max values. A general linear mixed model was used to determine if MEAN, MODE, and rMC: C were impacted by the tissues being imaged (renal zones [cortex and medulla], retroperitoneal fat of the kidneys, liver and spleen), sex or age groups (juvenile and sub-adults [< 15 years], adults [15–40 years] and geriatric elephants [> 40 years]) [26, 27]. Individual elephant was included in the model as the random variable, while tissue, sex and age groups were included as fixed variables. Akaike’s information criterion (AIC) was used to assess model fit. A change in model size by > 10% was used to assess fit. A least significant difference (LSD) test was used to further characterize differences in MEAN, MODE, and MC: C ratios between tissue regions.

When differences between tissues were not different by sex or age, the data were pooled to establish reference intervals using the ASVCP guidelines [28]. Outliers were screened using the Tukey test and Dixon’s outlier range statistic. Reference intervals for normally distributed data were calculated using both a parametric method and a robust method, according to the guidelines established by the Clinical and Laboratory Standards Institute (CLSI) [29]. Confidence intervals were set at 90% and were determined around both the lower and upper limits of the reference interval using the bootstrap method. The central 95th percentile of non-normally distributed data was determined using non-parametric methods established by the CLSI. Confidence intervals for the lower and upper limits could not be determined for this data set due to the number of samples being less than 120. MedCalc 20.115 (MedCalc Software, Ostend, Belgium) was used to compile the reference intervals.

## Results

**Fig. 3:**
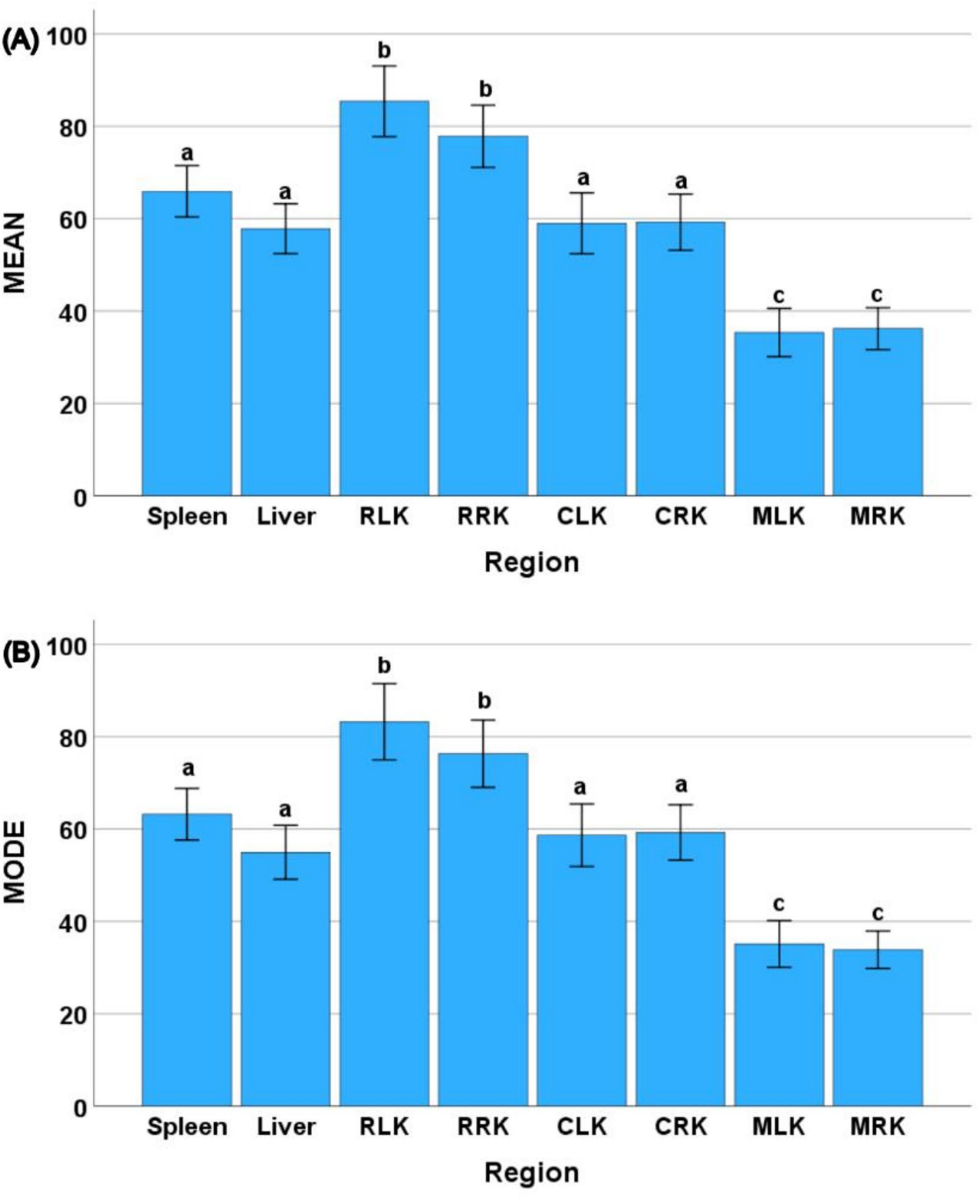
The bar charts illustrating the average (± SEM) of MEAN (**A**) and MODE (**B**) of gray-scale derives from ultrasonographical images of spleen, liver, retroperitoneal fat of the kidneys (left [RLK] and right [RRK] side), renal cortex (left [CLK] and right [CRK] side) and renal medulla (left [MLK] and right [MRK] side) from Asian elephants (*N* = 49). Statistically significant differences (*p* < 0.001) in MEAN (A) or MODE (B) values between organ regions were assessed using a linear mixed model with least significant difference (LSD) post-hoc tests, as indicated by letters above the error bars

**Fig. 4:**
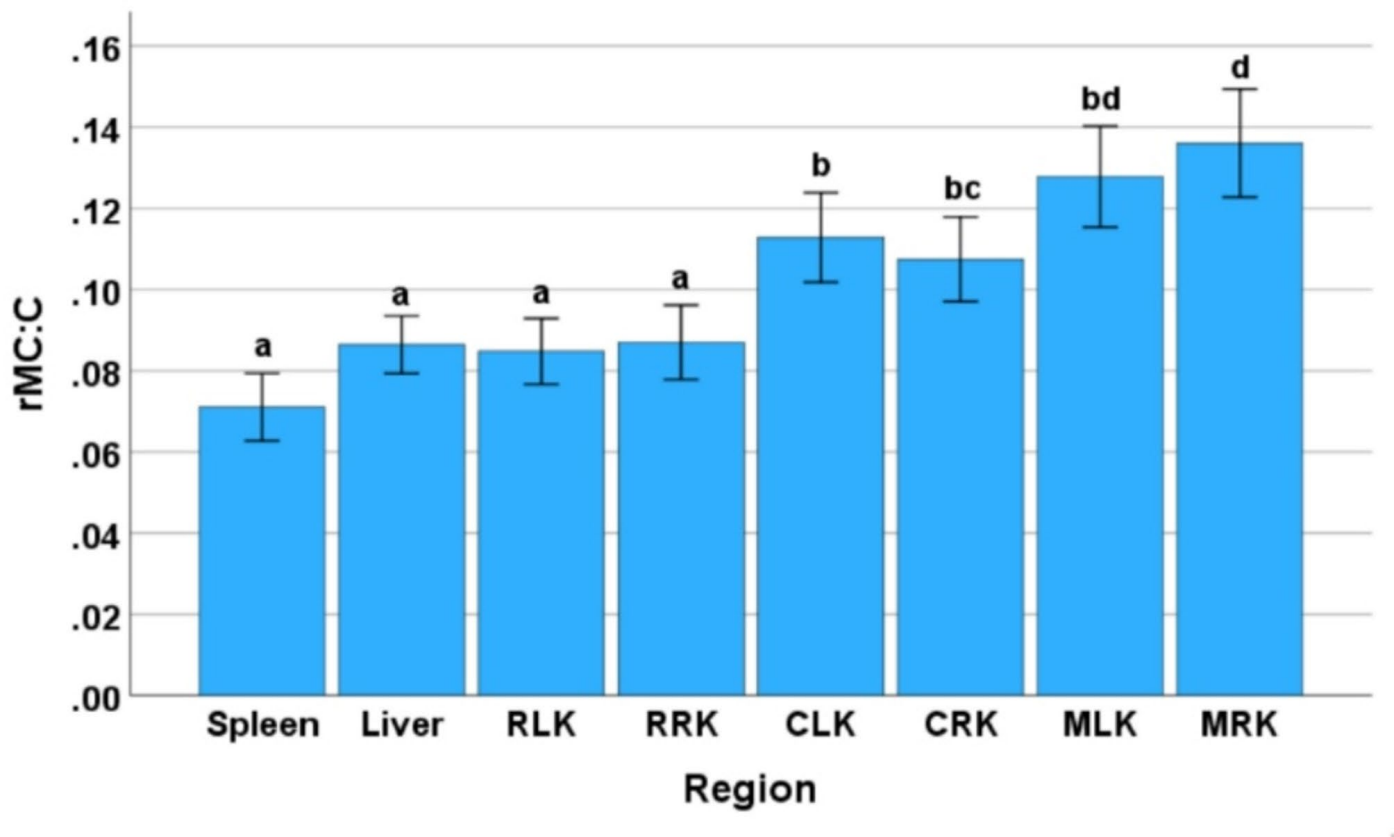
The bar charts illustrating the average (± SEM) of the ratio between MODE COUNT and COUNT (rMC: C) of gray scale derived from ultrasonographical images of spleen, liver, retroperitoneal fat of the kidneys (left [RLK] and right [RRK] side), renal cortex (left [CLK] and right [CRK] side) and renal medulla (left [MLK] and right [MRK] side) from Asian elephants (*N* = 49). Statistically significant differences (*p* < 0.001) in MEAN (A) or MODE (B) values between organ regions were assessed using a linear mixed model with least significant difference (LSD) post-hoc tests, as indicated by letters above the error bars

Average distances of 22.82 ± 4.7 cm (left kidney) and 23.64 ± 5.58 cm (right kidney) from the spine and 21.59 ± 5.2 cm (left kidney) and 21.64 ± 5.13 cm (right kidney) from the left and right iliac wing were recorded during probe placement. Eight locations of interest were captured, and 3 ROI were selected for GLH assessment. Based on the MEAN, MODE, and rMC: C values of all regions, the results indicated that retroperitoneal fat of the left kidney (RLK) was the most hyperechoic location (highest MEAN and MODE), followed by retroperitoneal fat of the right kidney (RRK) and spleen. The most hypoechoic location was the left renal medulla (MLK), right renal medulla (MRK) and liver respectively. The right renal medulla (MRK), left renal medulla (MLK) and left renal cortex (CLK) exhibited the most homogenous echotexture (highest rMC: C values), respectively. The right renal medulla (MRK) was the most heterogenous followed by the left renal medulla (MLK) and left renal cortex (CLK). There were significant differences between the different tissues (MEAN [F = 70.52, *P* < 0.001]; MODE [F = 61.59, *P* < 0.001]; and rMC: C [F = 23.91, *P* < 0.001]) for all three parameters (Figs. 3 and 4). There were no significant differences in the MEAN and MODE values between the spleen, liver and renal cortex. The MEAN and MODE values of the retroperitoneal fat were significantly higher (*P* < 0.001) than those of all other regions, while the renal medulla exhibited the lowest values. No significant difference was found for rMC: C values between the spleen, liver and retroperitoneal fat. The rMC: C values of the spleen, liver and retroperitoneal fat were significantly lower (*P* < 0.001) than those of the renal cortex and medulla. There were no significant differences in MEAN, MODE or rMC: C values between age groups (MEAN [F = 0.589, *P* = 0.559]; MODE [F = 0.696, *P* = 0.504]; and rMC: C [F = 0.25, *P* = 0.78]) or sex (MEAN [F = 0.111, *P* = 0.741]; MODE [F = 0.296, *P* = 0.589]; and rMC: C [F = 1.69, *P* = 0.2]). Tables 1 and 2 represent references intervals for normal and non-normally distributed data by each tissue site, respectively.

**Table 1:** Reference intervals for MEAN, MODE and R MC: C for each tissue type. Because these data were found to Meet the assumption of normality, they are reported by the mean, standard deviation, minimum-maximum values, and 95% reference intervals using both the normal and robust methods

Parameter	Mean	SD	Min-Max	Outliers	95th Percentile Reference Interval (normal distribution)	90% CI for Lower Limit (normal distribution)	90% CI for Upper Limit (normal distribution)	95th Percentile Reference Interval (robust method)	90% CI for Lower Limit (robust method)	90% CI for Upper Limit (robust method)
Spleen MEAN (*n* = 49)	65.9	19.3	27.8-111.4	none	28-103.8	20.1–35.9	95.9-111.7	26.4-105.3	19.2–34.8	97.9-112.8
Spleen MODE (*n* = 49)	63.2	19.5	27–113	none	25.1-101.3	17.1–33	93.4-109.3	23-102.5	15.3–31	94.3–110
Liver MEAN (*n* = 44)	53.1	12.9	24.9–87	91.7,93.4,95.3, 105.9,110.2	27.8–78.4	22.2–33.4	72.8–83.9	26-78.7	20.3–32.7	72.7–84.5
Liver MODE (*n* = 44)	49.6	12.9	27–82	93,95,95,111,114	24.2–75.1	18.6–29.8	69.5–80.7	22.5–75.5	16.9–29.4	70.5–81.2
Liver rMC: C (*n* = 49)	0.09	0.02	0.04–0.15	none	0.04–0.13	0.03–0.05	0.12–0.14	0.03–0.14	0.02–0.04	0.12–0.15
Retroperitoneal Left Kidney MEAN (*n* = 49)	85.4	26.6	29.8-155.9	none	33.3-137.5	22.3–44.2	126.6-148.4	28.9-138.1	18.6–39.6	130-149.5
Retroperitoneal Left Kidney MODE (*n* = 49)	83.2	28.7	27–158	none	26.9-139.5	15.2–38.7	127.7-151.3	21.8-139.3	10.9–34.1	125.2-152.7
Left Renal Cortex rMC: C (*n* = 49)	0.11	0.04	0.04–0.21	none	0.04–0.19	0.02–0.05	0.17–0.2	0.03–0.19	0.01–0.04	0.17–0.21
Left Renal Medulla rMC: C (*n* = 48)	0.12	0.04	0.06–0.22	0.27	0.05–0.2	0.03–0.06	0.18–0.22	0.04–0.21	0.03–0.06	0.18–0.22
Retroperitoneal Right Kidney MEAN (*n* = 49)	77.8	23.5	25.2-129.8	none	31.8-123.8	22.2–41.4	114.2-133.4	28.6-124.2	19.9–38.1	113.3-134.6
Right Renal Cortex MEAN (*n* = 45)	54.5	14.2	19.3–87.3	99.6, 110.4,119.1,121.2	26.7–82.3	20.6–32.8	76.2–88.3	24.5–82.3	18.6–30.5	75.1–89.3
Right Renal Cortex MODE (*n* = 45)	54.9	15.1	20–85	98,106,110,121	25.3–84.4	18.9–31.8	77.9–90.8	21.5–86	16.7–28.7	77.1–92.3
Right Renal Medulla MEAN (*n* = 47)	34.3	13	11-68.3	80.3,82.4	8.8–59.7	3.4–14.2	54.3–65.1	5.3–60.7	0.6–10.9	53-66.1
Right Renal Medulla rMC: C (*n* = 49)	0.14	0.05	0.06–0.24	none	0.05–0.23	0.03–0.07	0.2–0.24	0.04–0.23	0.02–0.06	0.21–0.25

**Table 2:** Reference intervals for MEAN, MODE and R MC: C for each tissue type. Because the data were not found to Meet the assumption of normality, they are reported by the median, interquartile range, minimum-maximum values, and 95% reference intervals using both the non-parametric and robust methods

Parameter	Median	IQR	Min-Max	Outliers	95th Percentile Reference Interval (Non-parametric method)	95th Percentile Reference Interval (robust method)	90% CI for Lower Limit (robust method)	90% CI for Upper Limit (robust method)
Spleen rMC: C (*n* = 45)	0.06	0.05–0.08	0.03–0.12	0.13,0.14,0.17, 0.17	0.03–0.11	0.02–0.10	0.006–0.03	0.09–0.11
Retroperitoneal Left Kidney rMC: C (*n* = 46)	0.07	0.06–0.09	0.05–0.12	0.14,0.14,0.2	0.05–0.12	0.04–0.12	0.03–0.04	0.1–0.12
Left Renal Cortex MEAN (*n* = 49)	53.5	45.7–74.5	23.9-111.3	none	24.5-110.9	6.8-102.9	-0.5-16.1	90.9-115.2
Left Renal Cortex MODE (*n* = 48)	53	44–70	24–109	116	24.7-108.5	7.5–99.9	1.6–17.3	89.6-111.9
Left Renal Medulla MEAN (*n* = 45)	27.8	22.8–40.5	10.4–62.8	84.77, 82.94, 73, 70.61	10.5–62.3	0.4–57.4	-4.1–6.8	50.1–64.9
Left Renal Medulla MODE (*n* = 48)	33	24-44.5	9–77	83	9.2–75.4	-1.8–64.6	-9.5-4.2	56.5–74
Retroperitoneal Right Kidney MODE (*n* = 49)	192	192–192	75–300	75,75,75,108, 108,108,108,147, 147,147,300	75–273	N/A	N/A	N/A
Retroperitoneal Right Kidney rMC: C (*n* = 48)	0.08	0.06–0.1	0.04–0.15	0.21	0.04–0.15	0.03–0.14	0.02–0.04	0.12–0.15
Right Renal Cortex rMC: C (*n* = 49)	0.1	0.08–0.13	0.05–0.21	none	0.05–0.21	0.03–0.18	0.01–0.04	0.16–0.20
Right Renal Medulla MODE (*n* = 48)	29	25.5–45.2	10–62	80	10.8–60.2	4.3–57.3	-0.04-9.5	50.2–63.4

## Discussion

Renal disease is a reported cause of morbidity and mortality in elephants. However, definite diagnosis often only occurs from post-mortem examination [4]. Geriatric populations are speculated to have renal issues, which brings upon the need for an early, non-invasive detection tool for renal condition assessment, and transcutaneous ultrasound has been found to be a useful non-invasive antemortem tool for assessing renal structure, size and abnormalities in a number of animal species; however, prior to this study, there had been no report on the use of this tool to assess the kidneys of Asian elephants.

When comparing ultrasound scans of the kidneys, spleen and liver in Asian elephants through the GLH technique, it is observed that the renal medulla appears more hypoechoic and homogenous than the renal cortex. The spleen and liver demonstrate comparable echogenicity to the renal cortex, but it exhibits less homogeneity in comparison. Additionally, the retroperitoneal fat displays the highest echogenicity, whereas the spleen is identified as the least homogeneous organ. The results align partially with what has been outlined in the existing literature. In humans, the kidneys are hypoechoic or isoechoic compared with the normal liver or spleen [30]. The normal renal cortex is usually hypoechoic or sometimes isoechoic to that of the liver or spleen. Studies in cats [31, 32], horses and mules [11] have also found the renal cortex to be more hyperechoic than the renal medulla and hypoechoic or isoechoic when compared to the liver and spleen. In our study, the echogenicity of the renal cortex and liver were not significantly different, similar to the study in cats [32].

From the present study, no significant differences in echogenicity or echotexture were found between the age groups or the sexes. Previous studies in humans found that liver echogenicity increased in aging livers with conditions such as steatosis [33], patients with extreme obesity and adolescent age and male gender [34]. We speculate that this contrasts with our results due to our sample population consists of healthy animals from one facility with less husbandry variability compared to independent humans. This suggests that healthy Asian elephants, regardless of age or sex, have similar echogenicity and echotexture calculated from GLH. We set our inclusion criteria for healthy elephants based on the results of a complete blood count, past medical history, and absence of structural abnormalities during ultrasound. Therefore, the results from this study can serve as a baseline for transcutaneous ultrasound in healthy elephants.

The primary objective of this study was to determine whether transcutaneous ultrasound could be used to assess the kidneys of Asian elephants, and to determine if there were innate distinctions in the echogenicity and echotexture of the kidneys compared with other organs (liver, spleen, fat) and between different age groups and sexes. Based on these results, the next logical step is to apply this ante-mortem tool to Asian elephants with renal disease, where hypo or hyper echogenicity might be observed in hydronephrosis or fibrosis, as indicated in renal equine ultrasonography [35]. In human medicine, GLH has been used to diagnose infectious hydronephritis [36]. Future studies investigating elephants with illnesses should focus on changes in echogenicity or echotexture through GLH.

There were several limitations associated with this study that should be addressed. First, the study did include a small sample size and a lack of diverse representation. We had nine juveniles to sub-adult elephants in comparison to 22 geriatric elephants. Even though we did not find any significant difference between age groups, a larger sample size may provide different results. Another limitation was that the elephants in this study were managed in captivity in free contact, so they were accepting of the methods carried out for the study. In this captive setting, they are trained to tolerate medical procedures such as phlebotomy, ophthalmic examination, and abdominal ultrasonography. This approach allowed us to conduct ultrasonography examination solely under the direction of the mahout without the need for restraint, sedation or anesthesia. It is important to note that not all elephants can be managed this way. Chemical restraint may be necessary for untrained animals. For elephants managed in protected contact or with hands-off management, training the elephant to allow transcutaneous ultrasound, though challenging, is possible and should be attempted. The parts of body i.e. ROI, should be placed to the window of the protected contact wall, which would allow the sonographer to perform renal ultrasonography. Another limitation is the difficulty in visualizing organs. It is subjectively noted by the authors that the size and body condition of the elephant factor in how challenging it can be to locate target organs. For example, it was easier to identify organs in younger elephants because of their lower body condition scores and weight. Elephants with high BCS have excess fat, which can impede image acquisition, particularly of the kidneys [37]. In our study, elephants had an interquartile range of 3.5-4 for BCS on a scale of 1–5. The narrow IQR shows that the population does not have a widespread representation of BCS. We cannot conclude in this study if BCS influenced our results, and future studies comparing low to high BCS are recommended. Routine preventative examinations are usually performed annually and including transcutaneous ultrasound for ongoing monitoring of echogenicity and echotexture may aid in the diagnosis of renal disease. An increase in renal echogenicity may warrant further investigation into underlying renal disease (e.g. acute kidney injury, chronic kidney disease, glomerular disorders) [17].

Another challenge is the large size of elephants, which does not allow visualization of multiple organs in one window, or even the entirety of the kidney itself. This is where quantitative analysis is essential because adjustments in gain and depth when locating organs might alter the sonographer’s perception of echogenicity and echotexture.

## Conclusion

Transcutaneous ultrasound was a useful and non-invasive tool for renal assessment and visualization of the kidneys, liver and spleen in captive Asian elephants. Quantitative assessment with GLH in a healthy population revealed baseline values of echogenicity and echotexture of different regions for comparison and as a reference for detecting structural pathologies. The findings in this study provide species-specific information for clinicians to utilize as a baseline for the evaluation of the kidneys, liver and spleen of Asian elephants. Further investigation is needed to identify detectable differences between individuals with disease and healthy subjects.

## Data Availability

The data and materials are available from the corresponding author upon reasonable request.
